# Validation of the care transition measure in multi-ethnic South-East Asia in Singapore

**DOI:** 10.1186/1472-6963-12-256

**Published:** 2012-08-16

**Authors:** Anu Birla Bakshi, Shiou-Liang Wee, Charlene Tay, Loong-Mun Wong, Ian Yi-Onn Leong, Reshma A Merchant, Nan Luo

**Affiliations:** 1 Health Promotion Board, Singapore, Singapore; 2 Agency for Integrated Care, Singapore, Singapore; 3 Tan Tock Seng Hospital, Singapore, Singapore; 4 National University Hospital, Singapore, Singapore; 5 Saw Swee Hock School of Public Health, National University of Singapore, Singapore, Singapore

## Abstract

**Background:**

The 15-item Care Transition Measure (CTM-15) is a measure for assessing the quality of care during transition from the patients’ perspective. The purpose of this study was to test the psychometric properties of the CTM-15 and CTM-3 (a 3-item version of the CTM-15) in Singapore, a multi-ethnic urban state in South-east Asia.

**Methods:**

A consecutive sample of patients was recruited from two tertiary hospitals. The subjects or their proxies were interviewed 3 weeks after discharge from hospital to home in English or Chinese using the CTM-15 questionnaire. Information about patients’ visit to emergency department (ED), non-elective rehospitalisation for the condition of index hospitalisation, and care experience after discharge was also collected from respondents. Psychometric properties of CTM-15 and CTM-3 based on the five-point response scale (i.e. strongly disagree, disagree, neutral, agree, and strongly agree) and the three-point response scale (i.e. [strongly] agree, neutral, and [strongly] disagree) were tested for English and Chinese versions separately. Internal consistency reliability was assessed using Cronbach’s alpha and construct validity was tested with *T*-test or Pearson’s correlation by examining hypothesised association of CTM scores with ED visit, rehospitalisation, and experience with care after discharge. Exploratory factor analysis was performed to examine latent dimensions of CTM-15.

**Results:**

A total of 414 (proxy: 96.1%) and 165 (proxy: 84.8%) subjects completed the interviews in English and Chinese, respectively. Cronbach’s alpha values of the different CTM-15 versions ranged from 0.81 to 0.87. In contrast, Cronbach’s alpha values of the CTM-3 ranged from 0.42 to 0.63. Both CTM-15 and CTM-3 were correlated with care experience after discharge regardless of survey language or response scale (Pearson’s correlation coefficient: 0.36 to 0.46). Among the English-speaking respondents, the CTM-15 and CTM-3 scores based on both the three- and five-point response scales discriminated well between patients with and without ED visits or rehospitalisation for their index condition. Among Chinese-speaking respondents, no difference in CTM scores was observed between patients with and without ED visits or patients with and without rehospitalisation. The English and Chinese versions of the CTM-15 items demonstrated a similar 4-factor structure representing general care plan, medication, agreement on care plan, and specific care instructions.

**Conclusions:**

The care transition measure is a valid and reliable measure for quality of care transition in Singapore. Moreover, the care transition measure can be administered to proxies using a simpler response scale. The discriminatory power of the Chinese version of this instrument needs to be further tested in future studies.

## Background

Care transition, or the movement of patients from one healthcare setting to another, is a concern of many healthcare systems because it affects the overall quality of patients’ care. It has been well documented that inadequate discharge planning and/or patients’ poor self-management following discharge from hospitals increased avoidable rehospitalisation and the use of emergency room [1-5]. It is not surprising that leading healthcare organisations for quality and safety such as the Joint Commission and The National Quality Forum (USA) are emphasising high-quality care transition [6-12].

An important element in the measurement of quality of transition is patients’ experience [13,14], as patients and caregivers are often the only common link across various care providing settings. In 2005, the American Institute of Medicine recommended research and development of measures to address patients’ experience during care transitions [15].

The Care Transition Measure (CTM-15) is, to the best of our knowledge, the only available measure of quality of care during transition, from the patients’ perspective [16-19]. It was designed to measure the overall care transition experience and not merely the hospital discharge phase [20]. Studies have shown that the CTM-15 is a valuable tool for health system performance evaluation by measuring the quality of care delivered across settings [17,20]. Recently, a shorter version of the CTM-15 comprising 3 of the original CTM items (CTM-3) [16,21] was endorsed by the National Quality Forum [22]. The CTM-3 closely approximates the CTM-15 in measuring the quality of care transition and has an advantage of minimising cost and response burden for assessing quality of care [16]. Both the CTM-15 and CTM-3, however, have been validated in patient populations in the US and Israel only [23].

The purpose of our study was to test the psychometric properties of the CTM-15 and CTM-3 in Singapore, a multi-ethnic urban state in South-east Asia. The quality of Singapore’s healthcare services was regarded high by both the World Health Organization [24] and Singaporeans [25]. Although it is not in the form of national health insurance, Singapore’s healthcare system has successfully achieved the goal of providing good and affordable basic medical care to all Singaporeans. Nevertheless, as a fast aging country with high public hospitals bed occupancy, Singapore is also facing the challenge of growing concerns over quality of care transition [26,27]. Therefore, there is a need to have a measure of care transition to assess the quality of care of patients in transition between care settings in Singapore. As the majority of the Singaporean population speaks either English or Chinese, we assessed both the English and Chinese versions of this instrument. We also compared the effects of three-point and five-point Likert response scales (currently used in the CTM) on the psychometric properties of the measures. This analysis was for maximising the usefulness of the instrument as fewer response choices pose less administrative and cognitive burden to respondents.

## Methods

### Study setting and sampling

Patients who were being discharged from two tertiary public hospitals in Singapore: National University Hospital (NUH) and Tan Tock Seng Hospital (TTSH) between January to June 2009 were recruited prospectively as subjects for this survey study. Inclusion criteria were inpatients aged 50 years and above, hospital care by disciplines of general medicine, general surgery, orthopedics, or geriatric medicine, and home residence upon discharge. At the time of this study, both hospitals had in place a care transition program that was implemented in collaboration with the Agency for Integrated Care (AIC) in June (NUH) and September (TTSH) 2008. AIC was a state funded national agency which coordinated referrals and care for people who require long term care. Dedicated care coordinators employed and trained by AIC and based at the respective hospitals coordinated discharge planning and care transition for patients from the hospitals to community settings. In order to increase the representativeness of the study sample, we recruited equal number of subjects from the two participating hospitals and those who were and were enrolled by the care coordination program. The National Healthcare Group Ethics Board, which had research ethics purview of both hospitals, approved the study.

### Data collection

Before discharge, patients were informed about the survey and consent was taken. Patients were contacted by a research assistant 3 weeks after their discharge from the hospital for an interview over the phone between February to July 2009 using the CTM-15 questionnaire. Interviews were conducted in English or Chinese based on respondents’ preference. In case a patient was not contactable, too weak to be interviewed, or had language barrier, a caregiver who started to look after the patient before discharge, if available, was interviewed as a proxy of the patient. The survey also sought information about patients’ demographics, length of stay before discharge, visit to emergency department, non-elective rehospitalisation for the condition of index hospitalisation, and experience with care after discharge.

### Instrument

The CTM-15 is a 15-item measure of perceived quality of care transition. The measure is based on a tested conceptual framework containing items relating to patients’ critical understanding, importance of preferences, management preparation and existence of a written and understandable care plan [20]. All questions use a five-point Likert response scale comprising ‘strongly disagree’, ‘disagree’, ‘neutral’, ‘agree’ and ‘strongly agree’. A single total score ranging from 0 to 100 can be calculated from the CTM-15, with higher scores indicating better care transition. In this study the English (US) version and its Chinese translation were used. The Chinese version was developed by a single forward translation of the English CTM conducted by one of the Chinese-speaking investigators of this study.

The CTM-3 comprises three of the CTM-15 items: (i) patients’ understanding of their medications; (ii) the extent to which patient preferences are taken into account in deciding what the patient’s health-care needs will be on discharge; and (iii) patients’ understanding of their responsibilities in managing their health [28]. Hence, the CTM-3 score can be calculated from the administration of the CTM-15. In this study, the CTM-3 score was transformed to the same range (0 to 100) of the CTM-15 score for the purposes of easy comparison.

A total of five statements were used to assess patients’ negative experience with care after discharge including (i) I felt like I really need to talk to a health professional but did not know whom to call; (ii) I was treated more like a number than a person; (iii) I went to a doctor or nurse who seemed to not understand my healthcare needs; (iv) I had difficulties getting the care I thought I needed; and (v) I found that people did not really care about me as a person. Those questions were used in a previous study for assessing the CTM-15 [17]. In the present study, answers to the five questions (yes = 0/no = 1) were used to calculate a total score, with higher scores indicating better experience.

### Statistical analysis

Descriptive analysis was performed to describe the study sample by calculating frequency for categorical variables and mean (standard deviation) for continuous variables. CTM item and total scores were described using mean (standard deviation) for easy comparison, although those were not interval data.

The internal consistency reliability of the CTM was assessed by Cronbach’s alpha. An alpha value of ≥ 0.7 was considered satisfactory [29]. Construct validity was assessed by testing *a priori* hypotheses. We hypothesised that patients who visited emergency department (ED) or were rehospitalised for the index condition after discharge would have lower CTM scores than those who did or were not (known-groups validity). The mean CTM score for patients with and without ED visits and patients with and without rehospitalisation were tested using two-sample t-tests. We also hypothesised that the CTM score would be at least moderately correlated with the experience score (convergent validity). For testing the criterion validity of the CTM-3, we hypothesised that the CTM-15 and CTM-3 would be highly correlated. Correlation was assessed by Pearson’s correlation coefficient. A correlation coefficient < 0.35, 0.35 to 0.49, and 0.5 to 1.0 were considered weak, moderate, and strong, respectively [29].

The English and Chinese language versions of the CTM-15 and CTM-3 were assessed separately. We also examined the validity and reliability of the CTM-15 and CTM-3 based on the three-point Likert response scale. We did this analysis because a three-point response scale such as disagree/neutral/agree would be easier to both administer and answer. Previous studies suggested that the three-point response scale is preferable to the five-point response scale for use in health-status questionnaires for respondents who are illiterate [30] or with intellectual disability [31]. For testing a hypothetical three-point response scale, we assigned ‘strongly (dis)agree’ and ‘(dis)agree’ the same coding as if respondents had answered on a three-point response scale (i.e. disagree/neutral/agree).

In order to examine the latent constructs measured by the CTM-15 items, we performed exploratory factor analysis. Factors with an eigenvalue > 1.0 were consider important and retained in a Varimax rotation analysis. Items with a factor loading > 0.5 were used to name the resultant factors.

All analyses were performed with SPSS for Windows (version 13) and all statistical tests were 2-tailed with a significance level of 0.05.

## Results

A total of 600 patients or their caregivers were interviewed. The overall participation rate was 58%. The main reason for refusal was lack of interest in the study. After excluding 21 respondents who were interviewed in Malay, 579 patients or their caregivers who were interviewed in English (n = 414) or Chinese (n = 165) with no missing data on the CTM-15 questions were included in data analysis. Table 1 summarises the basic demographic information of the participants of the study. It can be seen that the vast majority of respondents was proxies. English-speaking and Chinese-speaking respondents had similar characteristics except that higher proportion of respondents were patients themselves among those who were interviewed in Chinese (15.2%) than in English (3.9%) (p <0.01). Distribution of CTM item scores are displayed in Table 2. For both English and Chinese respondents, CTM-3 scores were lower than CTM-15 scores, and CTM scores based on the 5-point response scale were lower than those based on the 3-point response scale.

**Table 1:** Characteristics of respondents

**Variable**		**Total n = 579**	**English-speaking n = 414**	**Chinese-speaking n = 165**	P-value^*^
Gender, n (%)	Male	239 (41.3)	168 (40.6)	71 (43)	0.593
	Female	340 (58.7)	246 (59.4)	94 (57)	
Age (years)	Mean (SD)	77.16 (8.93)	77.23 (8.68)	76.98 (9.52)	0.761
	Range	50 - 103	50 - 103	51 - 99	
Identity of respondent, n (%)	Patient	41 (7.1)	16 (3.9)	25 (15.2)	0.001
	Spouse	212 (36.6)	147 (35.5)	65 (39.4)	
	Child (in-law)	286 (49.4)	219 (52.9)	67 (40.6)	
	Relative/other	40 (6.9)	32 (7.7)	8 (4.8)	
Length of stay (days)	Mean (SD)	10.75 (9.07)	11.03 (9.56)	10.04 (7.67)	0.245
	Range	1 - 75	1 - 75	2 - 44	
Enrolment in care coordination program, n (%)	Yes	293 (50.6)	214 (51.7)	79 (47.9)	0.408
	No	286 (49.4)	200 (48.3)	86 (52.1)	
Hospital, n (%)	NUH	285 (49.2)	210 (50.7)	75 (45.5)	0.252
	TTSH	294 (50.8)	204 (49.3)	90 (54.5)	
ED visits, n (%)	Yes	83 (14.3)	63 (15.2)	20 (12.1)	0.349
	No	496 (85.7)	351 (84.8)	145 (87.9)	
Rehospitalisation, n (%)	Yes	90 (15.5)	69 (16.7)	21 (12.7)	0.220
	No	489 (84.5)	345 (83.3)	144 (7.3)	

* Chi-square test or independent 2-sample *t*-test.

**Table 2:** Means and standard deviations of CTM item and total scores measured on 5-point and 3 point response scales

**CTM item**	**English CTM on 5-point response scale**		**English CTM on 3-point response scale**		**Chinese CTM on 5-point response scale**		**Chinese CTM on 3-point response scale**	
	**Mean**	**SD**	**Mean**	**SD**	**Mean**	**SD**	**Mean**	**SD**
1. Agreed health goals and means	62.0	29.5	66.2	47.0	57.3	29.3	61.8	47.8
2. Preferences deciding health needs*	68.1	25.5	79.0	38.5	61.7	26.7	70.6	43.8
3. Preferences deciding where needs met	69.9	23.9	82.6	35.8	64.7	25.2	75.8	40.4
4. Had information needed for self-care	67.5	27.4	75.7	42.4	66.1	27.2	73.9	43.7
5. Understand how to manage health	73.4	24.3	84.7	35.1	68.8	24.9	78.8	40.6
6. Understand signs and symptoms	53.5	29.9	51.6	48.7	49.5	29.3	45.2	48.5
7. Understand medications’ purpose	77.4	19.0	91.8	24.5	76.1	19.6	90.9	26.6
8. Understand how to take medications	79.5	17.0	94.2	19.7	78.0	17.2	93.6	21.5
9. Understand medications’ side effects*	45.2	26.3	38.6	44.9	47.4	28.6	42.4	47.4
10. Had written list appointments and lists	71.0	16.7	88.3	28.5	73.6	17.5	91.8	26.6
11. Had written care plan	37.0	23.1	24.9	41.7	38.8	25.8	28.2	43.2
12. Understand what makes better or worse	67.0	27.6	76.2	42.0	59.1	27.5	65.2	47.0
13. Understand care responsibilities *	72.5	24.8	83.7	36.4	67.4	23.8	80.0	39.7
14. Confident knew how to manage	74.1	20.8	88.4	29.9	70.6	19.5	86.1	32.5
15. Confident could do what needed	72.6	22.5	85.9	32.4	68.0	22.4	81.5	36.7
CTM-15 total score	66.0	14.7	74.1	19.8	63.1	15.4	71.1	22.9
CTM-3 total score	61.9	17.7	67.1	25.6	58.8	18.1	64.3	29.3

Note: item scores are coded as 0 = strongly disagree, 25 = disagree, 50 = neutral, 75 = agree, 100 = strongly agree for the 5-point scale and as 0 = strongly disagree or disagree, 50 = neutral, 100 = strongly agree or agree for the 3-point scale; * CTM-3 items.

### CTM-15

The Cronbach’s alpha values of the CTM-15 scores bases on the five-point and three-point response scale were 0.87, 0.81 for the English version and 0.82, 0.85 for the Chinese version, respectively.

Among English-speaking respondents, patients who did not report ED visits for the index condition had higher mean CTM-15 scores than those who reported ED visits; similarly, patients who reported no rehospitalisation for the index condition had higher CTM-15 scores than those who reported rehospitalisation (Table 3). However, such differences in CTM-15 scores were not observed in the Chinese-speaking subgroup (Table 3). For example, the mean CTM-15 scores based on the 5-point scale were 60.9 and 63.2 (p > 0.05, two-sample test) for patients with and without ED visits, respectively. There was moderate correlation between the CTM-15 and the experience scale, regardless of survey language and response scale (correlation coefficient: 0.39 to 0.46, p < 0.001 for all, Table 3).

**Table 3:** Association of CTM score with ED visits, rehospitalisation, and experience with care

		**English CTM**						**Chinese CTM**					
		**Mean**	**SD**	P-value^*^	**Mean**	**SD**	P-value^*^	**Mean**	**SD**	P-value^*^	**Mean**	**SD**	P-value^*^
		**CTM-15 based on 5-point response scale**			**CTM-15 based on 3-point response scale**			**CTM-15 based on 5-point response scale**			**CTM-15 based on 3-point response scale**		
ED visit for similar condition	Yes	62.1	19.7	0.020	67.0	24.8	0.002	60.9	19.1	0.492	65.5	26.5	0.249
	No	66.8	13.5		75.4	18.5		63.4	14.9		71.8	22.4	
Rehospitalisation for similar condition	Yes	62.8	19.2	0.041	67.9	24.2	0.004	62.8	18.2	0.908	69.4	25.7	0.719
	No	66.7	13.5		75.4	18.6		63.2	15.0		71.3	22.5	
Correlation with EXP score, *Pearson's r*		0.397		<0.001	0.392		<0.001	0.461		<0.001	0.426		<0.001
		**CTM-3 based on 5-point response scale**			**CTM-3 based on 3-point response scale**			**CTM-3 based on 5-point response scale**			**CTM-3 based on 3-point response scale**		
ED visit for similar condition	Yes	56.1	23.0	0.004	57.4	30.3	0.001	56.3	21.9	0.498	58.3	32.7	0.329
	No	63.0	16.4		68.9	24.3		59.2	17.6		65.2	28.8	
Rehospitalisation for similar condition	Yes	56.9	22.8	0.009	58.7	30.1	0.003	58.7	21.8	0.977	63.5	33.6	0.887
	No	62.9	16.3		68.8	24.4		58.9	17.6		64.5	28.7	
Correlation with EXP score, P*earson's r*		0.399		<0.001	0.389		<0.001	0.378		<0.001	0.356		<0.001

* Independent 2-sample test.

The English and Chinese versions of the CTM-15 items measured on the five-point scale demonstrated a similar 4-factor structure. Based on item loadings, the four factors were general care plan, medication, agreement on care plan, and specific care instructions (Table 4). Factor analysis of the 15 CTM items based on the three-point scale showed similar factor structures (data not shown).

**Table 4:** Factor loadings of the CTM-15 items measured on the 5-point scale after Varimax rotation

	**English CTM-15**				**Chinese CTM-15**			
	**Factor 1**	**Factor 2**	**Factor 3**	**Factor 4**	**Factor 1**	**Factor 2**	**Factor 3**	**Factor 4**
1. Agreed health goals and means	0.537	0.540				0.654		
2. Preferences deciding health needs		0.847				0.885		
3. Preferences deciding where needs met		0.853				0.884		
4. Had information needed for self-care	0.744				0.546			
5. Understand how to manage health	0.745				0.654			
6. Understand signs and symptoms				0.665				0.655
7. Understand medications’ purpose			0.823				0.765	
8. Understand how to take medications			0.884				0.735	
9. Understand medications’ side effects				0.627			0.566	
10. Had written list appointments and lists			0.572				0.671	
11. Had written care plan				0.782				0.733
12. Understand what makes better or worse	0.683			0.330				0.547
13. Understand care responsibilities	0.808				0.647			
14. Confident knew how to manage	0.839				0.856			
15. Confident could do what needed	0.803				0.869			
Variance explained	67.3%				67.7%			

### CTM-3

The Cronbach’s alpha value of the CTM-3 based on the five-point response scale and three-point response scale was 0.58 and 0.42 for the English version, and 0.63 and 0.57 for Chinese version, respectively. The correlation between the CTM-3 and CTM-15 was 0.89 and 0.87 for English and Chinese versions, respectively, when the five-point response scale was used; the correlation was 0.83 and 0.76 for the English and Chinese version, respectively, when they were scored on the three-point response scale.

Similar to the results for CTM-15, the CTM-3 scores discriminated well between patients with and without ED visits or rehospitalisation among English-speaking respondents; however, the CTM-3 scores were not different between patients reporting and not reporting ED visits or rehospitalisation in the Chinese-speaking group (Table 3). The correlation between the CTM-3 and experience scale was moderate for both language groups and response scales (correlation coefficient: 0.36 to 0.40, p < 0.001 for all, Table 3).

## Discussion

We found that the psychometric measurement properties of the CTM were generally satisfactory, although variations existed between different versions of this instrument. Generally, the long form is more reliable than the short form and English version is more valid than the Chinese version. The English version of the CTM-15 is the most valid and reliable measure of care transition. It exhibited adequate internal consistency and was able to discriminate between patients with and without ED visits or rehospitalisation and converged with reported experience with care post-discharge. These results were consistent with those in previous studies [16,17,23]. Also, three of the four factors underlying the CTM items in our study were similar to those observed in the US and Israel populations [16,23].

Most of participants in our study were not patients but their proxies. This was because most patients designated a family member to answer the interview questions for them. Reasons for using a proxy included caregivers knowing better than patients about care-related matters, patients too weak to be interviewed, and patients having language barriers. Generally, using proxies to assess quality of care is a limitation because proxies and patients themselves may have very different perception of the care during transition. Nevertheless, it is necessary to use proxies when patients are not well enough to be the assessors. Moreover, it makes good sense to ask for the opinions of family members when it comes to assessment of quality of care from care users’ perspective in Singapore. Singapore is a small city country dominated by Asian culture and Singaporeans are very involved in the medical and long term care of their family members. Family members, particularly spouses and children not only provide informal care, but influence or even make medical and long term care decisions for the patient and also contribute to the healthcare costs. Singapore’s unique healthcare system allows contribution of healthcare costs from family members using individuals’ compulsory savings, which strengthens family bonds. Hence, it is not unexpected that the majority of our respondents were family members (Table 1). For this reason, a quality of care measure would be relevant and more valuable in Singapore with proxies’ assessment. The most important contribution of our study is therefore the provision of evidence for the usefulness of CTM when it is administered to proxies. To the best of our knowledge, this is the first study that explored the extended application of this instrument to assessor other than patients themselves.

The Chinese CTM-15 and CTM-3 were similar to their English counterparts in psychometric properties but exhibited poor discriminatory power. There are several possible reasons for this result. First, the poor discriminatory power could be partly be attributed to sampling error. This is supported by the observation that less than thirty patients reported ED visits or rehospitalisation in the Chinese-speaking subgroup (Table 1). It is possible that those patients visited ED or were rehospitalised for reasons not related to poor care transition. Therefore, the Chinese CTM should be reevaluated in a larger sample of patients in the future. Second, it could also be attributed to respondents’ poor understanding of the CTM items. Although we did not collect information on education profile of the patients, Chinese-speaking patients generally had fewer years of formal education than English Speaking patients in Singapore [32,33] as English has been the primary language in education. Since the statements used in the CTM questionnaire are relatively long, a larger proportion of Chinese-speaking respondents might not comprehend the statements fully, which in turn affected the discriminatory power of the CTM. Another possible reason for poor understanding is suboptimal wording of the Chinese CTM which was the product of a single forward translation of the English CTM. Certain Chinese CTM items might not be accurate or clear. Nevertheless, this may not be a serious issue; factor analysis of English and Chinese CTM items resulted in similar latent constructs, suggesting no dramatic difference in response patterns between the two language groups. Third, Chinese-speaking respondents may have idiosyncratic response styles due to culture reasons. A previous study found that a visual analog scale for self-assessing overall health was not discriminative between Singaporean diabetics in differing health conditions [34]. Exact reason for this phenomenon is unknown. Hence, future studies using qualitative research methods such as in-depth interview or focus group discussion are needed to find out how respondents understand and respond to the CTM items, which will hopefully help us find ways to improve the performance of the instrument among Chinese-speaking persons.

It is interesting that the CTM scores based on the three-point response scale performed similarly well as those based on the originally five-point response scale. That means if the interviewer had offered only three response options (i.e. disagree, neutral, and agree) in the interviews, the resultant CTM scores would not be less reliable or discriminative. This result is not surprising as three-point response scales were found to be superior to five-point scales in some populations [35]. This is a useful finding as it suggests that the CTM can be administered in a more economical and easy-to-answer way.

It was not unexpected that the CTM-3 demonstrated worse internal consistency than the CTM-15. Internal consistency increases with increasing number of items. Although the reliability of the CTM-3 was suboptimal, it was highly correlated with the CTM-15 scores and exhibited good construct validity in English-speaking respondents. Therefore, the CTM-3 can be used as a substitute for the CTM-15 when administration burden is a concern or in large-scale studies where large sample sizes can compensate its suboptimal reliability.

There are some limitations in the study. First, our findings have limited generalisability. Factors that limit generalisability include relatively low response rate, all patients aged 50 years or older, half of the study sample enrolled in a care coordination program, most respondents being proxies, and some proxies possibly not experiencing the entire care transition process. However, our study for the first time demonstrated that the CTM was valid and reliable in proxies. Second, that we administered the CTM at the time when ED visits and rehospitalisation had occurred may have introduced response bias to the study. That is, those who had experienced ED visits or rehospitalisation might ascribe those undesirable events to care transition, which in turn intensified their memory of negative experience during discharge including those experienced during the discharge process. Arguably, it may be more appropriate to administer the CTM soon right after the hospital discharge (with the discretion of the problem we discussed next) and obtain information about ED visits and rehospitalisation from administrative records or inquire at a later time so that the respondents’ answers to the CTM questions are more clearly disassociated from ED visits or readmission events. Third, although genuine, the reports from some older patients may not be reliable. A previous study suggested delirium, including periods of altered perception, is highly prevalent among the elderly recently discharged to a post-acute care facility from hospital [30]. However, this should not be a big concern as the vast majority of our respondents were their caregivers.

## Conclusion

The care transition measure is a valid and reliable measure for quality of care transition in Singapore. Moreover, the care transition measure can be administered to proxies using a simpler response scale. The discriminatory power of the Chinese version of this instrument needs to be further tested in future studies.

## Abbreviations

CTM: Care transition measure; ED: Emergency department.

## Competing interests

The authors declare that they have no competing interests.

## Authors’ contribution

ABB and NL conceptualized the study, conducted the data analysis, interpreted results, and drafted the manuscripts. SLW revised the manuscript and is responsible for the content of the paper. All other authors planned the study, facilitated data collection, read and approved the final manuscript.

## Pre-publication history

The pre-publication history for this paper can be accessed here:

http://www.biomedcentral.com/1472-6963/12/256/prepub
